# Associations Between Depression Symptom Severity and Daily-Life Gait Characteristics Derived From Long-Term Acceleration Signals in Real-World Settings: Retrospective Analysis

**DOI:** 10.2196/40667

**Published:** 2022-10-04

**Authors:** Yuezhou Zhang, Amos A Folarin, Shaoxiong Sun, Nicholas Cummins, Srinivasan Vairavan, Linglong Qian, Yatharth Ranjan, Zulqarnain Rashid, Pauline Conde, Callum Stewart, Petroula Laiou, Heet Sankesara, Faith Matcham, Katie M White, Carolin Oetzmann, Alina Ivan, Femke Lamers, Sara Siddi, Sara Simblett, Aki Rintala, David C Mohr, Inez Myin-Germeys, Til Wykes, Josep Maria Haro, Brenda W J H Penninx, Vaibhav A Narayan, Peter Annas, Matthew Hotopf, Richard J B Dobson, RADAR-CNS Consortium

**Affiliations:** 1 Department of Biostatistics & Health Informatics Institute of Psychiatry, Psychology and Neuroscience King's College London London United Kingdom; 2 Institute of Health Informatics University College London London United Kingdom; 3 NIHR Biomedical Research Centre at South London and Maudsley NHS Foundation Trust London United Kingdom; 4 Health Data Research UK London University College London London United Kingdom; 5 NIHR Biomedical Research Centre at University College London Hospitals NHS Foundation Trust London United Kingdom; 6 Janssen Research and Development LLC Titusville, NJ United States; 7 Department of Psychological Medicine Institute of Psychiatry, Psychology and Neuroscience King's College London London United Kingdom; 8 School of Psychology, University of Sussex Falmer United Kingdom; 9 Department of Psychiatry Amsterdam UMC location Vrije Universiteit Amsterdam Netherlands; 10 Mental Health Program Amsterdam Public Health Research Institute Amsterdam Netherlands; 11 Teaching Research and Innovation Unit Parc Sanitari Sant Joan de Déu Fundació Sant Joan de Déu Barcelona Spain; 12 Centro de Investigación Biomédica en Red de Salud Mental Madrid Spain; 13 Faculty of Medicine and Health Sciences Universitat de Barcelona Barcelona Spain; 14 Department of Psychology Institute of Psychiatry, Psychology and Neuroscience King's College London London United Kingdom; 15 Department of Neurosciences Center for Contextual Psychiatry Katholieke Universiteit Leuven Leuven Belgium; 16 Faculty of Social Services and Health Care LAB University of Applied Sciences Lahti Finland; 17 Center for Behavioral Intervention Technologies Department of Preventive Medicine Northwestern University Chicago, IL United States; 18 South London and Maudsley NHS Foundation Trust London United Kingdom; 19 H Lundbeck A/S Copenhagen Denmark

**Keywords:** depression, gait, mobile health, mHealth, acceleration signals, monitoring, wearable devices, mobile phones, mental health

## Abstract

**Background:**

Gait is an essential manifestation of depression. However, the gait characteristics of daily walking and their relationships with depression have yet to be fully explored.

**Objective:**

The aim of this study was to explore associations between depression symptom severity and daily-life gait characteristics derived from acceleration signals in real-world settings.

**Methods:**

We used two ambulatory data sets (N=71 and N=215) with acceleration signals collected by wearable devices and mobile phones, respectively. We extracted 12 daily-life gait features to describe the distribution and variance of gait cadence and force over a long-term period. Spearman coefficients and linear mixed-effects models were used to explore the associations between daily-life gait features and depression symptom severity measured by the 15-item Geriatric Depression Scale (GDS-15) and 8-item Patient Health Questionnaire (PHQ-8) self-reported questionnaires. The likelihood-ratio (LR) test was used to test whether daily-life gait features could provide additional information relative to the laboratory gait features.

**Results:**

Higher depression symptom severity was significantly associated with lower gait cadence of high-performance walking (segments with faster walking speed) over a long-term period in both data sets. The linear regression model with long-term daily-life gait features (*R^2^*=0.30) fitted depression scores significantly better (LR test *P*=.001) than the model with only laboratory gait features (*R^2^*=0.06).

**Conclusions:**

This study indicated that the significant links between daily-life walking characteristics and depression symptom severity could be captured by both wearable devices and mobile phones. The daily-life gait patterns could provide additional information for predicting depression symptom severity relative to laboratory walking. These findings may contribute to developing clinical tools to remotely monitor mental health in real-world settings.

## Introduction

Depression affects the lives of over 300 million people worldwide [1] and is associated with many adverse outcomes, including decreased quality of life, loss of occupational function, disability, premature mortality, and suicide [2-5]. While early treatment can be effective and prevent more serious adverse outcomes [6], more than half of depressed people do not receive timely treatment [7,8]. Current questionnaire-based depression assessments may be affected by recall bias and may not be able to collect dynamic information [9,10]. Therefore, several recent studies have attempted to explore the associations between depression and changes in individuals’ behaviors using mobile technologies [11].

Changes in gait are essential manifestations of depression [12,13]. The main hypothesis linking gait with depression is a bidirectional interaction between the brain motor system and cortical and subcortical structures, which are related to emotions and cognitive functions [14-16]. Many studies have explored the relationships between depression and gait characteristics based on “gold-standard” laboratory walking tests. Longer gait cycles, reduced stride length, and slower gait cadence were observed in participants with depression compared with healthy controls, which have been consistently shown in several studies [17-25]. Other gait abnormalities such as reduced gait force [21], increased double support time [22], reduced swing time variability [23], slumped postures [24], and increased body sway [25] have been reported, but with less consistency across studies.

Laboratory gait tests are hard to be applied in real-world settings because of the need for expensive equipment (eg, video camera and force plates), specialized laboratories, and the inconvenience of wearing sensors on the knees and ankles, for example [14,26]. Some researchers have suggested that people’s daily-life activity characteristics should have stronger links to their health conditions than laboratory tests [27-29]. Therefore, it is necessary to monitor and evaluate daily-life walking using efficient methods.

In recent years, several studies have used mobile technologies to measure daily-life walking patterns and explored their associations with depression. However, most of these studies only measured the number of cumulative steps of daily-life walking [30-32], which is more related to individuals’ mobility and physical activity than to gait patterns (eg, gait cadence and gait force). To our knowledge, there have been only a few studies exploring the associations between daily-life gait patterns and depression directly. Adolph et al [33] found that depressed participants had reduced walking speed, reduced vertical up-and-down movements, and more slumped postures compared with controls by placing two accelerometers on the participant’s trunk and right leg for 2 days [33]. However, wearing multiple sensors on the body may not be suitable for long-term monitoring. With the development of sensors, the mobile phone provides a cost-effective, continuous, and unobtrusive means to measure individuals’ behaviors, including daily walking. Therefore, the mobile phone may be a potential tool for long-term gait monitoring.

The aim of this study was to explore the value of daily-walking monitoring for improving the evaluation of depression symptom severity. Our first objective was to design and extract gait features from raw acceleration signals to describe the characteristics of daily walking. The second objective was to explore the associations between gait features and depression symptom severity, and to test whether these associations could be captured by different acceleration devices. The third objective was to test whether daily-life walking could provide additional information for predicting depression relative to laboratory walking. To achieve the second and third objectives, we performed our analyses on two ambulatory data sets, the Long Term Movement Monitoring (LTMM) and Remote Assessment of Disease and Relapse–Major Depressive Disorder (RADAR-MDD) data sets [34,35], with acceleration signals collected by a wearable device and mobile phone, respectively. Importantly, the LTMM data set contains data related to both laboratory and daily walking, which could address the third study objective.

## Methods

### Data Sets

#### LTMM Data Set

The LTMM data set includes demographics (age and gender), depression scores (15-item Geriatric Depression Scale [GDS-15] [36]), and raw acceleration signals (100 Hz) of laboratory walking tests and 3-day activities for 71 elderly adults [34], which can be downloaded at PhysioNet [37]. Participants were included if they did not have any cognitive or gait/balance disorders [34]. Participants were asked to walk at a self-selected and comfortable speed for 1 minute in the laboratory while wearing a 3-axis accelerometer on their lower back [34]. The GDS-15 questionnaire contains 15 easy-to-understand, yes/no format questions, which is suitable for depression screening in the older population [38,39]. After the laboratory walking test, all participants were asked to wear the accelerometer for the next 3 consecutive days to record daily activities [34].

#### Ethics Considerations

RADAR-MDD was conducted per the Declaration of Helsinki and Good Clinical Practice, adhering to principles outlined in the National Health Service (NHS) Research Governance Framework for Health and Social Care (2nd edition). Ethical approval has been obtained in London from the Camberwell St Giles Research Ethics Committee (REC reference 17/LO/1154), in Spain from the CEIC Fundació Sant Joan de Deu (CI PIC-128-17), and in the Netherlands from the Medische Ethische Toetsingscommissie VUms (METc VUmc registratienummer 2018.012–NL63557.029.17).

#### RADAR-MDD Data Set

The EU research program RADAR-MDD aimed to investigate the utility of mobile technologies for the long-term monitoring of participants with depression in real-world settings [35,40]. Adult participants with a depression history were included in the study if they did not meet the following criteria: (1) have other psychiatric disorders (eg, bipolar disorder, schizophrenia, and dementia), (2) have received treatment for drug or alcohol use in the past 6 months, (3) a major medical diagnosis that affects daily activities, and (4) pregnancy [35]. A detailed study protocol was published previously [35]. In this study, we used a subset of RADAR-MDD data collected from a study site in the United Kingdom (King’s College London [KCL]) between November 2017 and April 2021, because the KCL site was the only site to acquire ethical approval for collecting the phone’s acceleration signals. We hereafter denote this subset as the RADAR-MDD-KCL data set for convenience. The phone’s acceleration signals were collected at 50 Hz and uploaded to an open-source platform, RADAR-base [41]. The participants’ depression symptom severity was assessed by the 8-item Patient Health Questionnaire (PHQ-8) [42] self-reported through mobile phones every 2 weeks. A patient advisory board comprising service users co-developed the study. They were involved in the choice of measures, timing, and issues of engagement, and have also been involved in developing the analysis plan.

### Step Detection Algorithm

**Figure 1:**
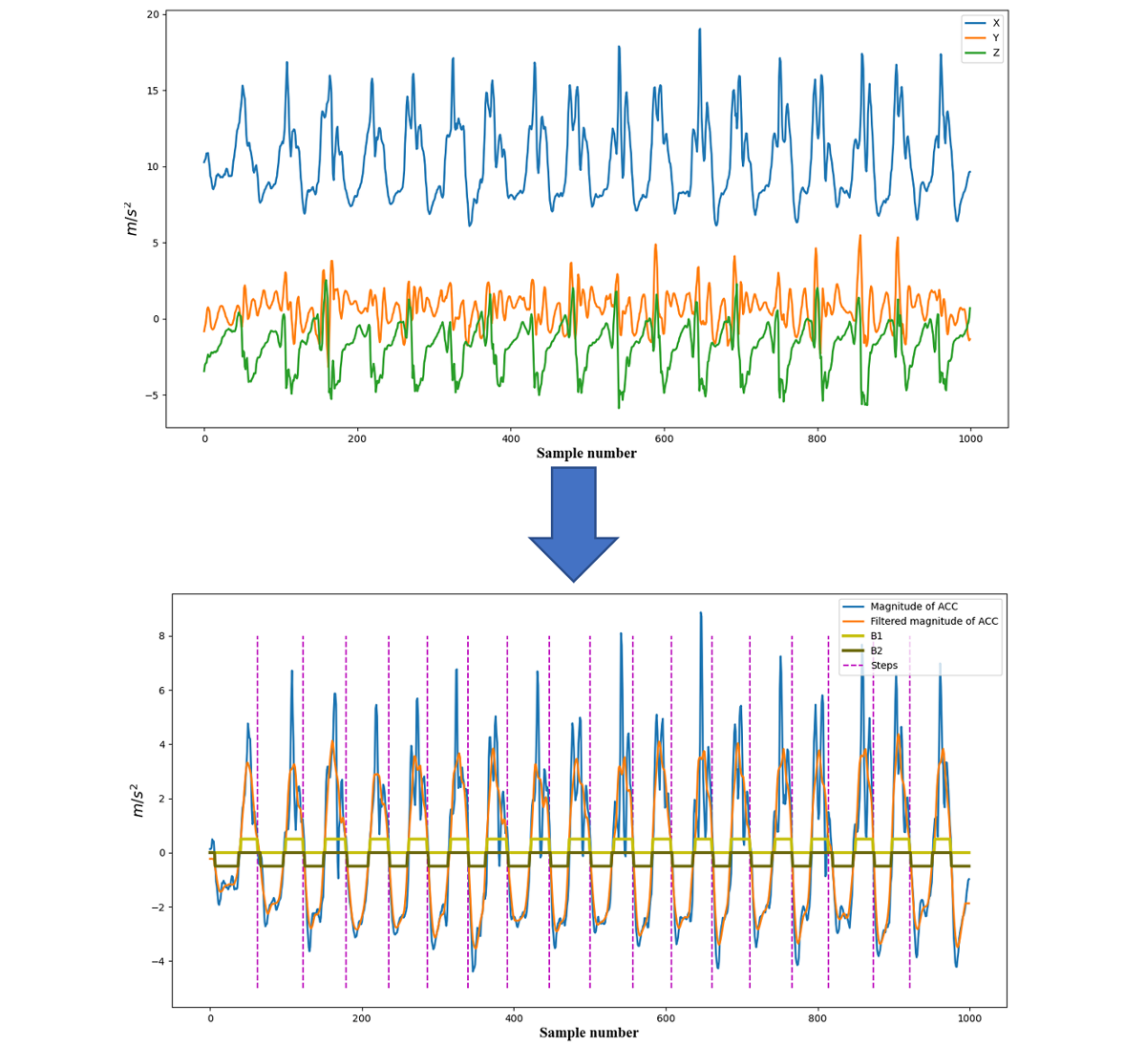
Step detection algorithm. ACC is the 3-axis acceleration signal; B1 and B2 are two series calculated by thresholds to detect walking swing and stance phase, respectively; and pink dashed lines represent the detected steps.

Since we needed to respectively detect steps on the acceleration signals collected by wearable devices and mobile phones, we chose to use the step detection algorithm [43], which was based on mobile phones (Figure 1). Given a segment of 3-axis acceleration signals (*x_i_, y_i_, z_i_*), the magnitude of the acceleration of the segment of acceleration signals was calculated to combine 3D signals to a single series, *r_i_*, where 
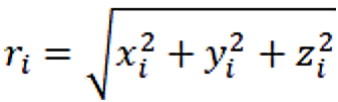
. The magnitude of the acceleration signals does not depend on the orientation and tilt of the mobile phone during walking [43]. Subsequently, *r_i_* was filtered by a weighted moving-average filter to remove noise (Equation 1, *w*=150 milliseconds). Next, the filtered *r_i_* was subtracted by the mean of *r̄_i_* to make *r̄_i_* symmetric to the x-axis. We calculated two new series, *B*1_i_ and *B*2_i_, based on two thresholds to detect the walking swing phase and stance phase, respectively (see Equations 2 and 3). If a swing phase ends and a stance phase starts, we can identify a step that occurred. The formal detection rule of a step *S_i_* at sample *i* is that the following two conditions must be satisfied: (1) a change from –0.5 to 0 in B1 (*B*1*_i_*=0 and *B*1*_i_*_–1_=0.5); (2) there is at least one detection of *B*2=–0.5 in a window of size *w*=150 milliseconds in sample *i* (*Min*(*B*2*_i_*_:_*_i_*_+_*_w_*)=–0.5).



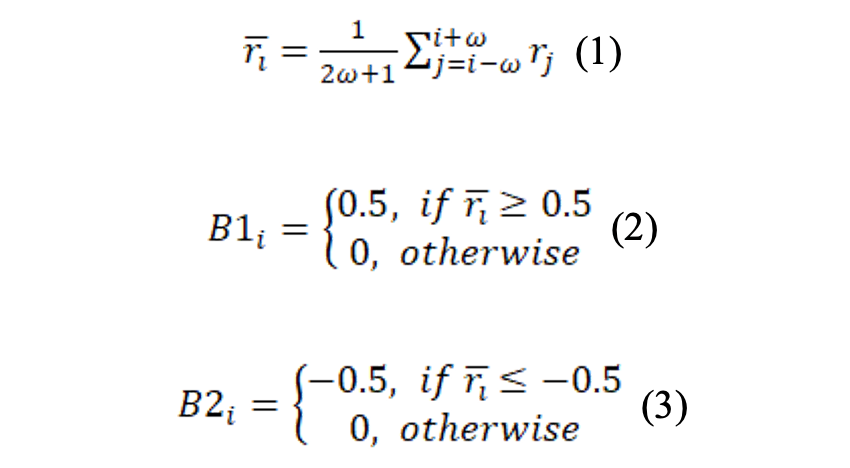



Then, the gait cycle series could be derived by calculating time intervals between consecutive steps, which was denoted as *Cycles.* During each gait cycle, the amplitude from the peak to the valley of the magnitude of the acceleration signals was used to reflect the gait force of each step. The force of all steps in the given acceleration signal was denoted as the series *Force.*

### Feature Extraction

#### Feature Window Size

Since the PHQ-8 score is used to estimate depression symptom severity for the past 2 weeks [42], we extracted gait features from a 14-day time window prior to each PHQ-8 record from the RADAR-MDD-KCL data set. For the LTMM data set, we extracted gait features from 3-day activities to link daily-life walking with the GDS-15 score.

#### Step Detection Window and the Continuous Walking Segment

**Figure 2:**
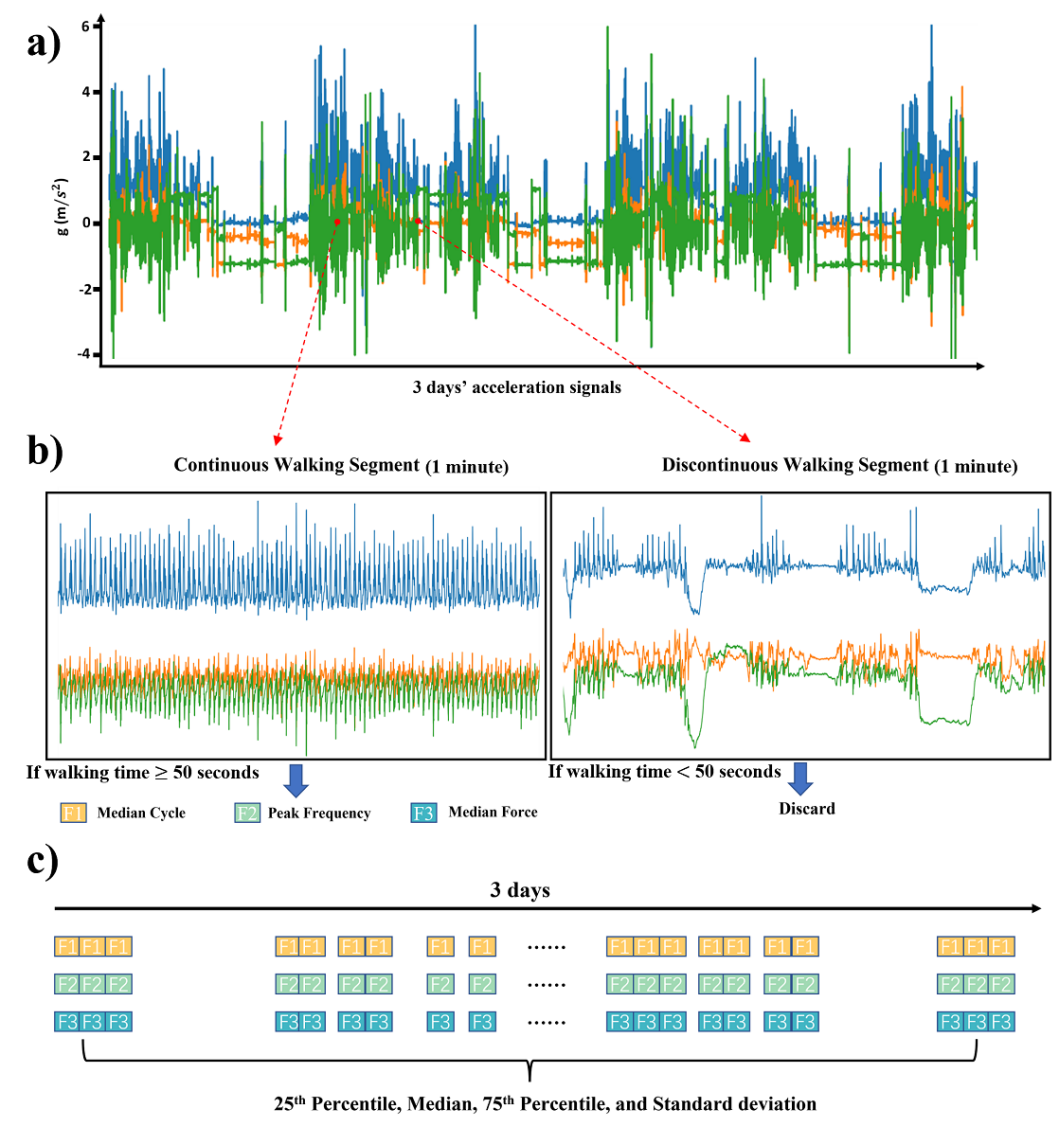
Schematic diagram of long-term gait feature extraction for the Long-Term Movement Monitoring data set. (a) Three-axis acceleration signals of 3 consecutive days; (b) examples of continuous and discontinuous walking segments and three short-term gait features (definitions in Table 1) were extracted from each continuous walking segment; (c) long-term gait feature extraction: 25th percentile, median, 75th percentile, and standard deviation of short-term gait feature values of all continuous walking segments over 3 days for each participant.

Daily-life walking in real-world settings is complex and contains some intermittent walking segments (such as walking in a crowded environment or a walking-rest transition status). These intermittent walking segments may not fully reflect a participant’s normal walking patterns. Therefore, to distinguish between continuous and intermittent walking, we used a 1-minute sliding window [44] to detect steps from the long-term raw acceleration signals. If the participant was walking most of the time in this minute, we considered this minute as the continuous walking segment. Based on our experience, we set 50 seconds as the threshold for selecting the continuous walking segment; that is, the segment with more than 50 seconds of walking time (sum of all gait cycles in the minute) was selected for further analysis (Figure 2b).

#### Gait Features

##### Overview

The performance of walking varies over time due to several factors such as mood, energy, and environment. Therefore, the long-term gait features need to represent the distribution and variance of walking patterns over the feature window. We first extracted three short-term gait features from every detected continuous walking segment in the feature window. Then, for each short-term gait feature, we calculated four statistical second-order features (long-term features) across all values of continuous walking segments. In total, 12 long-term gait features were extracted in this study, and a summary of these features is shown in Table 1. A schematic diagram of long-term gait feature extraction is shown in Figure 2.

**Table 1:** Short-term and long-term gait features extracted and their short descriptions.

Gait feature		Description
**Short-term gait features**		
	Median cycle (seconds)	Median of gait cycles in the 1-minute walking segment
	Peak frequency (Hz)	Peak frequency in the PSD^a^ of the magnitude of 1-minute acceleration signals
	Median force (m/s^2^)	Median of gait force in the 1-minute walking segment
**Long-term gait features**		
	25th percentile of median cycle	25th percentile of median gait cycle values of all walking segments^b^
	50th percentile of median cycle	Median of median gait cycle values of all walking segments
	75th percentile of median cycle	75th percentile of median gait cycle values of all walking segments
	SD of median cycle	Standard deviation of median gait cycle values of all walking segments
	25th percentile of peak frequency	25th percentile of peak frequency values of all walking segments
	50th percentile of peak frequency	Median of peak frequency values of all walking segments
	75th percentile of peak frequency	75th percentile of peak frequency values of all walking segments
	SD of peak frequency	Standard deviation of peak frequency values of all walking segments
	25th percentile of median force	25th percentile of median gait force values of all walking segments
	50th percentile of median force	Median of median gait force values of all walking segments
	75th percentile of median force	75th percentile of median gait force values of all walking segments
	SD of median force	Standard deviation of median gait force values of all walking segments

^a^PSD: power spectral density (from 0.5 Hz to 3 Hz).

^b^All detected continuous walking segments (defined in the Methods section) in a feature window (3 days for the Long Term Movement Monitoring data set and 14 days for the Remote Assessment of Disease and Relapse–Major Depressive Disorder data set).

##### Short-Term Gait Features From the 1-Minute Continuous Walking Segment

Gait cadence and gait force are essential characteristics of walking. Gait cadence is the rate at which the individual feet contact the ground [45]. Gait force reflects the ground reaction force during walking [46]. For every continuous walking segment, the median of the gait cycle series (*Cycles*) was calculated to reflect the gait cadence of this minute from the time domain, which was denoted as *median cycle*. To assess the gait cadence from the frequency domain, the power spectral density (PSD) of walking was obtained by applying the fast Fourier transformation to the filtered magnitude (*r̄_i_*) of the acceleration signals of every continuous walking segment. The peak frequency [47] of the 0.5-3–Hz band (reflecting walking) [34] of the PSD was used to reflect the main rhythm of steps from the frequency domain, which was denoted as *peak frequency*. For gait force, we calculated the median of the *Force* series (*median force*) to represent the average power of all steps in the minute.

##### Long-Term Gait Features

For each of the short-term gait features (*median cycle, peak frequency,* and *median force*), we calculated four statistical second-order features (25th percentile, median, 75th percentile, and SD) from all detected continuous walking segments during a feature window.

Previous studies suggested that the extreme values of gait characteristics over the long term could reflect the optimal or worst walking performance of the participant, which could in turn reflect physical or mental conditions better than the median value [29]. Therefore, we used *25th percentile*, *median*, and *75th percentile* second-order statistics to represent three levels of walking performance (low, medium, and high) during a feature window. For example, faster walking during a feature window could represent *high-performance walking*, which may not be affected by other factors such as fatigue and the crowded environment. *High-performance walking* could be represented by the *75th percentile of peak frequency* and the *25th percentile of median cycle* in a feature window, which is expected to be closely associated with depression status. The variance of daily-life walking in a feature window was measured by the SD.

##### Laboratory Gait Features Extracted From Laboratory Walking Tests in the LTMM Data Set

We also extracted *median cycle, peak frequency,* and *median force* from the 1-minute acceleration signals of laboratory walking tests in the LTMM data set. For reading convenience, we denoted these as laboratory gait features.

#### Inclusive Criteria for Data Missingness in the RADAR-MDD-KCL Data Set

The raw acceleration signals were remotely collected by mobile phones in the RADAR-MDD-KCL study. Possibly due to the high battery consumption and network traffic for uploading the raw signal, the missing rate of acceleration signals was relatively high. To reduce the impact of missingness, a PHQ-8 period (14 days) included in this study should have at least 3 days (aligned with the LTMM data set) with more than 50% acceleration signals [48,49].

### Statistical Analyses

For the LTMM data set, Spearman coefficients [50] were calculated to assess associations between the GDS-15 score and gait features (3 laboratory gait features and 12 long-term gait features). As the data in the RADAR-MDD-KCL data set are longitudinal (repeated PHQ-8 measurements for each participant), a series of pairwise linear mixed-effects regression models [51] with random participant intercepts were performed to explore the association between the PHQ-8 score and each of the 12 long-term gait features (no laboratory tests were included in the RADAR-MDD-KCL data set). Age, gender, and the number of comorbidities (see Table S1 in Multimedia Appendix 1) were considered as covariates. The Benjamini-Hochberg method was used for multiple-comparison corrections in both data sets [52].

To test whether long-term gait features could explain additional data variance in depression scores relative to laboratory gait features, we built two nested multivariate linear regression models without and with long-term gait features for the GDS-15 score (denoted as Model A and Model B; Equations 4 and 5) in the LTMM data set. Specifically, predictors of Model A are age, gender, and the 3 laboratory gait features, while predictors of Model B are age, gender, the 3 laboratory gait features, and the 12 long-term gait features. The coefficient of determination (*R*^2^) was calculated for both models to estimate how much data variance was explained by predictors. Then, the likelihood ratio test [53] was used to test whether Model B fit the GDS-15 score better than Model A. Since the laboratory walking test was not included in the RADAR-MDD-KCL data set, the likelihood ratio test was only performed in the LTMM data set.

Model A: GDS-15=Age+Gender+3 laboratory gait features **(4)**

Model B: GDS-15=Age+Gender+3 laboratory gait features+12 long-term gait features **(5)**

## Results

### Data Summary

**Figure 3:**
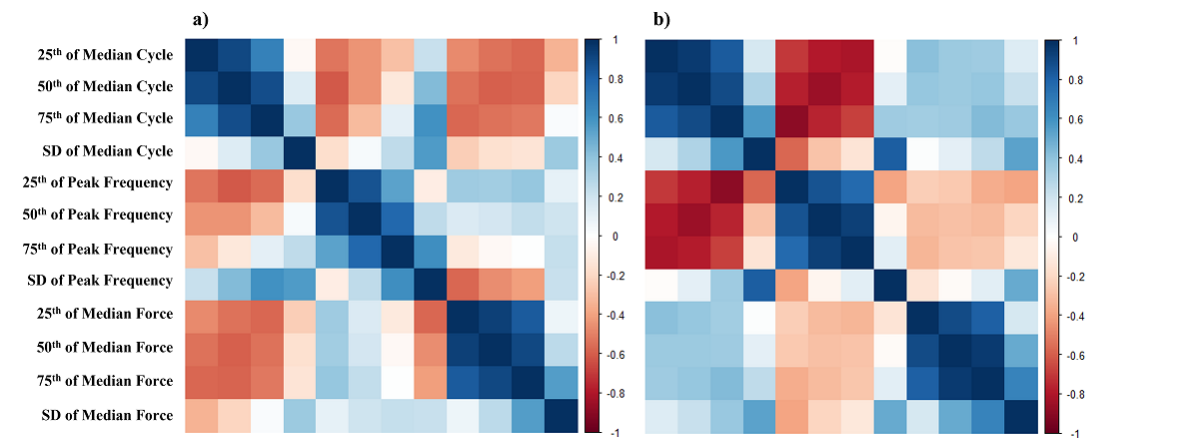
Heatmaps of correlations between 12 long-term gait features of the Long-term Movement Monitoring data set (a) and Remote Assessment of Disease and Relapse–Major Depressive Disorder King's College London data set (b).

The 71 participants in the LTMM data set have a mean age of 78.36 (SD 4.71) years with 18 (25%) participants having potential depressive disorders (GDS-15≥5) and 69.82 (SD 9.65) hours of acceleration signals per participant. The RADAR-MDD-KCL data set, according to the data inclusion criteria, contains 659 PHQ-8 records collected from 215 participants and corresponding 99,445 hours (average 463 hours per participant). The cohort in the RADAR-MDD-KCL data set has a mean age of 43.36 (SD 15.12) years with the majority being women (75%), and half of the PHQ-8 records indicated potential depression symptoms (PHQ-8≥10). The average missing rate of acceleration signals collected by phones in the RADAR-MDD-KCL data set (70.60%) was significantly higher than that of the acceleration signals collected by the wearable device in the LTMM data set (3.03%). A summary of the demographics, and distributions of depression scores and available acceleration signals for participants in the LTMM and the RADAR-MDD-KCL data sets is shown in Table 2. The heatmaps of correlations between the 12 long-term gait features of the LTMM and RADAR-MDD-KCL data sets are presented in Figure 3.

**Table 2:** Demographics and distributions of depression scores and available acceleration signals of participants in the two data sets.

Characteristic	LTMM^a^ (N=71)	RADAR-MDD-KCL^b^ (N=215)
Age (years), mean (SD)	78.36 (4.71)	43.36 (15.12)
Female, n (%)	46 (65%)	162 (75%)
Depression score, mean (SD)	GDS-15^c^: 3.18 (2.81)	PHQ-8^d^: 9.67 (5.84)
Potential depressive episode (GDS-15≥5) and PHQ-8≥10), n (%)^e^	18 (25%)	330 (50%)
Number of completed depression questionnaires^f^	71	659
Number of completed depression questionnaires per participant, mean (SD)	1 (0)	3.09 (2.76)
Length of total available acceleration signals (hours)	4817	99,445
Length of available acceleration signals (hours) for each GDS-15/PHQ-8 record^g^, mean (SD)	69.82 (9.65)	98.77 (105.20)
Average missing rate of acceleration signals (%)	3.03	70.60
Number of continuous walking segments^h^ detected from each GDS-15/PHQ-8 record, mean (SD)	73.48 (66.98)	113.24 (170.48)

^a^LTMM: Long Term Movement Monitoring.

^b^RADAR-MDD-KCL: subset of the Remote Assessment of Disease and Relapse–Major Depressive Disorder data set collected from King’s College London, United Kingdom.

^c^GDS-15: 15-item Geriatric Depression Scale.

^d^PHQ-8: 8-item Patient Health Questionnaire.

^e^Based on the total number of completed questionnaires.

^f^The RADAR-MDD-KCL data set has multiple PHQ-8 records for each participant, which was conducted every 2 weeks.

^g^We regarded acceleration signals in the 14 days before a PHQ-8 record. For the GDS-15 record, we considered acceleration signals of all 3-day activities after enrollment.

^h^Continuous walking segment was defined as 1-minute acceleration signals with at least 50 seconds of walking (see Methods section).

### Associations Between Gait Features and the GDS-15 Score in the LTMM Data Set

The Spearman correlations between the GDS-15 score and gait features (both laboratory and long-term gait features) in the LTMM data set are shown in Table 3. We found that a higher GDS-15 score was significantly correlated with a larger median of gait cycles, lower peak frequency, and smaller median gait force in the 1-minute laboratory walking test. For the long-term period, a higher GDS-15 score was significantly correlated with lower variance of gait force and slower cadence of high-performance walking and 75th percentile of peak frequency during 3-day activities.

**Table 3:** Spearman correlations between the 15-item Geriatric Depression Scale score and gait features, including laboratory and long-term gait features, in the Long-Term Movement Monitoring data set.

Feature^a^			*ρ*		*P* value^b^
**Laboratory gait features extracted from the 1-minute laboratory walking test**					
	Median cycle	0.39		.001	
	Peak frequency	–0.32		.01	
	Median force	–0.25		.04	
**Long-term gait feature extracted from 3-day activities**					
	25th percentile of median cycle	0.31		.01	
	50th percentile of median cycle	0.13		.29	
	75th percentile of median cycle	0.02		.86	
	SD of median cycle	–0.24		.06	
	25th percentile of peak frequency	–0.02		.85	
	50th percentile of peak frequency	–0.09		.45	
	75th percentile of peak frequency	–0.27		.03	
	SD of peak frequency	–0.12		.33	
	25th percentile of median force	0.02		.85	
	50th percentile of median force	–0.01		.98	
	75th percentile of median force	–0.10		.41	
	SD of median force	–0.30		.02	

^a^Definitions of gait features in this table are provided in Table 1 and the Methods section.

^b^*P* values were adjusted by the Benjamini-Hochberg method for correction of multiple comparisons.

### Associations Between Long-Term Gait Features and the PHQ-8 Score in the RADAR-MDD-KCL Data Set

The pairwise linear mixed-effects models performed in the RADAR-MDD-KCL data set revealed a significant and negative link between the PHQ-8 score and the gait cadence of *high-performance walking* during the 14 days before submitting PHQ-8 records. Specifically, the *25th percentile of median cycle* was positively associated with the PHQ-8 score; that is, for every increase of 0.1 seconds in the median gait cycle of *high-performance walking*, the PHQ-8 score increased by 0.606 points. Likewise, the *75th percentile of peak frequency* was negatively associated with the PHQ-8 score, indicating that a reduction of 0.1 Hz in the peak frequency of *high-performance walking* was associated with an increase of 0.26 PHQ-8 points. Other long-term gait features were not found to be significantly associated with the PHQ-8 score in the RADAR-MDD-KCL data set. A summary of all 12 linear mixed-effects regression models is provided in Table 4.

**Table 4:** Twelve pairwise linear mixed-effects models for exploring associations between long-term gait features and depression symptom severity (8-item Patient Health Questionnaire) in the RADAR-MDD-KCL data set.^a^

Long-term gait feature^b^	Estimate	SE	*df*	*t* value	*P* value^c^
25th percentile of median cycle	6.06	2.72	648.75	2.23	.03
50th percentile of median cycle	3.98	2.51	639.41	1.59	.11
75th percentile of median cycle	2.49	2.08	653.72	1.20	.23
STD of median cycle	2.87	4.41	631.11	0.65	.52
25th percentile of peak frequency	–1.50	1.02	656.44	–1.46	.15
50th percentile of peak frequency	–1.93	1.05	650.76	–1.83	.07
75th percentile of peak frequency	–2.62	1.01	634.70	–2.60	.01
SD of peak frequency	0.21	1.86	600.50	0.12	.91
25th percentile of median force	–0.57	2.24	637.46	–0.25	.80
50th percentile of median force	0.88	1.79	655.77	0.49	.62
75th percentile of median force	0.44	1.66	656.37	0.26	.79
SD of median force	2.05	3.78	602.90	0.54	.59

^a^RADAR-MDD-KCL: Subset of Remote Assessment of Disease and Relapse–Major Depressive Disorder collected from King’s College London.

^b^Definitions of daily-life gait features are provided in Table 1 and the Methods section.

^c^*P* values were adjusted by the Benjamini-Hochberg method for correction of multiple comparisons.

### Results of the Likelihood Ratio Test in the LTMM Data Set

The regression model with long-term gait features (Model B) achieved better performance (*R^2^*=0.30) than the model without long-term gait features (Model A) (*R^2^*=0.06). We found that the 12 long-term gait features extracted from 3-day activities could explain an extra 24% data variance (an increase of 0.24 in *R^2^*) of GDS-15 scores relative to the laboratory gait features and participants’ demographics. The likelihood ratio test showed that Model B fitted GDS-15 scores significantly better than Model A (*χ*^2^=32.91>*χ*^2^_0.05_(12), *P*=.001). The detailed results of the two nested regression models are shown in Table S2 of Multimedia Appendix 1.

## Discussion

### Principal Findings

This study retrospectively used two ambulatory data sets for exploring the associations between depression symptom severity and daily-life gait characteristics. We extracted 12 long-term gait features to describe the distribution and variance of gait cadence and force over a long-term period and link daily-life gait patterns with a self-reported depression score. The main findings of this study are (1) higher depression symptom severity is significantly associated with lower gait cadence of *high-performance walking* (faster walking in all continuous walking segments) over a long-term period; (2) long-term daily-life walking has the potential to provide additional information for predicting depression symptom severity relative to laboratory gait characteristics and demographics; and (3) wearable devices and mobile phones both have potential to capture the associations between daily gait and depression.

The results of Spearman correlations between laboratory gait features and the GDS-15 score in the LTMM data set are consistent with previous studies [17-25]; that is, the participants with more severe depression symptoms were more likely to have slower gait cadence (longer median of gait cycles and lower gait frequency) and smaller gait force in laboratory walking tests.

For daily-life walking, this study used the faster walking (*75th percentile of peak frequency* and *25th percentile of median cycle*) in all detected continuous walking segments to represent *high-performance walking* during a feature window (3 days for LTMM and 14 days for RADAR-MDD-KCL). Only gait cadence of *high-performance walking* was found to be significantly and negatively associated with depression symptom severity, whereas gait patterns under *medium/low-performance walking* were not significantly associated with the depression score. This finding was consistent in both the LTMM and RADAR-MDD-KCL data sets. A potential reason is that the walking performance in real-world scenarios may be affected by multiple factors (such as walking during the day or at night, walking under fatigue or walking after rest, and walking to a destination or navigating a crowded supermarket) [29]; therefore, the lower walking performance may not fully reflect the participant’s physical or mental conditions. Therefore, from the main finding of this study, we speculated that faster steps over a long-term period could represent the high performance of participants’ walking, which could be closely associated with their depression status.

In the LTMM data set, we found that the variance of gait force (*SD of median force*) in 3-day activities was significantly and negatively associated with the depression symptom severity, indicating that participants with higher depression symptom severity were likely to have relatively monotonous walking over 3 days. However, the feature was not significantly associated with the PHQ-8 score in the RADAR-MDD-KCL data set. One reason is that the magnitude (*r_i_*) (explained in the Step Detection Algorithm section) of the acceleration signals depends on the location of the accelerometers attached to the body [54]. As acceleration signals in the RADAR-MDD-KCL data set were collected by mobile phones, the variable locations of phones when attached to participants’ bodies (such as in the hand, handbag, and pocket) affected the magnitude of acceleration signals. Therefore, the magnitude of phone-collected acceleration signals cannot fully reflect the gait force.

Results of regression models and the likelihood test in the LTMM data set illustrated the importance of monitoring daily-life gait in real-world settings. Laboratory gait features and demographics in LTMM data only explained a small proportion of data variance of the GDS-15 score (*R*^2^=0.06), whereas long-term gait features extracted from 3-day activities could explain an extra 24% of data variance (*R*^2^=0.30). This finding supported that long-term daily-life walking has the potential to provide additional information for predicting depression symptom severity relative to laboratory gait characteristics and demographics. Further, this finding also indicated that the laboratory walking test may be affected by several factors such as subjective psychological factors and laboratory-controlled conditions, which may not fully reflect the condition of a participant’s mental health [27,29]. Since there were no laboratory tests in the RADAR-MDD-KCL data set, the comparison between laboratory gait features and long-term daily-life gait features was not performed in the RADAR-MDD-KCL. We will consider adding laboratory tests at enrollment in future digital depression studies.

### Limitations

Although we found that wearables and mobile phones have the potential to capture the associations between depression and daily-life gait patterns, both devices have some limitations. Wearables could collect relatively complete walking data; however, wearing sensors may not be suitable for long-term monitoring. Mobile phones could be used for long-term monitoring without user burden, but the missing rate of mobile phone acceleration signals is relatively high. The findings of this study support that the links between gait and depression could still be revealed from the limited and sparse daily-life walking acceleration signals. Missingness is a common challenge in remote digital studies [55], which may be caused by high battery consumption, network traffic for uploading the raw acceleration signals, and the Android operating system moderation of resources. According to the findings of this study, a possible solution to reduce missingness is uploading gait cycles instead of uploading raw acceleration signals in future long-term monitoring research. This is not difficult to implement, as most current smartphones have real-time step detection functions or apps [56,57]. Furthermore, the self-reported PHQ-8 data may be subject to recall bias. We may consider implementing ecological momentary assessments with passive gait data collection in future research.

The hyperparameters in step detection and feature extraction need further investigation. We considered using a 1-minute window size for step detection and 50 seconds for continuous walking segment selection based on previous studies [34,44] and our experience. The feature window sizes for the two data sets are different due to the different study designs. However, the optimal hyperparameters are still unclear and will be investigated in future research.

Gait features extracted in this study were simple and statistically based, which were used to illustrate the importance of daily walking in our initial analysis. More features such as nonlinear features will be considered in future research. 

Gait characteristics could be affected by some physical diseases, neurological disorders, and age [58-60]. Although none of the participants had any cognitive or gait/balance disorders in the LTMM data set and the number of comorbidities and age were considered as covariates in the RADAR-MDD-KCL data set, physical comorbidities and other comorbidities may have different impacts on the gait characteristics. We will consider a wider range of comorbidities and investigate them further in future research.

### Conclusion

In summary, the findings of this study showed that significant links between depression symptom severity and daily-life gait characteristics could be captured in different data sets and by different accelerometer devices. Long-term daily-life walking patterns could provide additional value for understanding depression manifestations relative to gait patterns in laboratory walking tests, which illustrated the importance of long-term gait monitoring. The gait cadence of high-performance walking in daily life has the potential to be an indicator for monitoring depression severity, which may contribute to developing clinical tools to remotely monitor mental health in real-world settings.

## Appendix

Multimedia Appendix 1 Tables S1-S2.

## Abbreviations

EFPIA: European Federation of Pharmaceutical Industries and Associations
FAST-R: Feasibility and Acceptability Support Team for Researchers
GDS-15: 15-item Geriatric Depression Scale
IMI: Innovative Medicines Initiative
LR: likelihood ratio
KCL: King’s College London
LTMM: Long Term Movement Monitoring
NHS: National Health Service
NIHR: National Institute of Health Research
PHQ-8: 8-item Patient Health Questionnaire
PSD: power spectral density
RADAR-CNS: Remote Assessment of Disease and Relapse–Central Nervous System
RADAR-MDD: Remote Assessment of Disease and Relapse–Major Depressive Disorder
