# Supplementary material for: Associations Between Depression Symptom Severity and Daily-Life Gait Characteristics Derived From Long-Term Acceleration Signals in Real-World Settings: Retrospective Analysis
Source: JMIR Mhealth Uhealth. 2022 Oct 4;10(10):e40667. doi: 10.2196/40667 (PMC9579931; doi:10.2196/40667)
Supplement: Multimedia Appendix 1 [file mhealth_v10i10e40667_app1.docx]

**Table S1.** The list of comorbidities that recorded at the enrollment session of the RADAR-MDD-KCL dataset^a^.

| Number | Comorbidity |
| --- | --- |
| 1 | Asthma |
| 2 | Chronic bronchitis |
| 3 | Other chest trouble |
| 4 | Diabetes |
| 5 | Depression |
| 6 | Stomach or other digestive disorder |
| 7 | Liver trouble |
| 8 | Kidney trouble |
| 9 | Rheumatoid arthritis |
| 10 | Osteoarthritis |
| 11 | Heart trouble |
| 12 | Cancer |
| 13 | High blood pressure |
| 14 | Multiple Sclerosis |
| 15 | Epilepsy/fits |
| 16 | Stroke |
| 17 | Other neurological trouble |
| 18 | Migraine |
| 19 | Back trouble |
| 20 | Other |

^a^ RADAR-MDD-KCL: A subset of the Remote Assessment of Disease and Relapse – Major Depressive Disorder dataset which was collected from King’s College London, United Kingdom.

**Table S2.** Results and performance of two nested linear regression models with (Model B) and without (Model A) long-term gait features in the Long-Term Movement Monitoring dataset.

| Feature^a^ | Model A | | | Model B | | |
| --- | --- | --- | --- | --- | --- | --- |
|  | Estimate | SE^b^ | P value | Estimate | SE | P value |
| (Intercept) | 9.49 | 11.57 | 0.42 | 17.36 | 22.98 | 0.45 |
| Age | -0.08 | 0.08 | 0.35 | -0.07 | 0.09 | 0.46 |
| Gender | -0.06 | 0.85 | 0.95 | -0.90 | 1.03 | 0.39 |
| Median Cycle | 0.02 | 0.08 | 0.77 | 0.05 | 0.13 | 0.67 |
| Peak Frequency | 0.38 | 1.92 | 0.85 | -0.43 | 2.10 | 0.84 |
| Median Force | -2.43 | 2.22 | 0.28 | -1.81 | 2.82 | 0.52 |
| 25th percentile of Median Cycle | —^c^ | — | — | 0.19 | 0.24 | 0.42 |
| 50th percentile of Median Cycle | — | — | — | -0.54 | 0.39 | 0.17 |
| 75th percentile of Median Cycle | — | — | — | 0.29 | 0.16 | 0.08 |
| STD of Median Cycle | — | — | — | -0.11 | 0.09 | 0.25 |
| 25th percentile of Peak Frequency | — | — | — | 8.54 | 7.07 | 0.23 |
| 50th percentile of Peak Frequency | — | — | — | -4.25 | 8.70 | 0.63 |
| 75th percentile of Peak Frequency | — | — | — | -8.33 | 4.30 | 0.06 |
| STD of Peak Frequency | — | — | — | 12.45 | 8.12 | 0.13 |
| 25th percentile of Median Force | — | — | — | 2.75 | 21.69 | 0.90 |
| 50th percentile of Median Force | — | — | — | 1.23 | 22.37 | 0.96 |
| 75th percentile of Median Force | — | — | — | 5.38 | 28.31 | 0.85 |
| STD of Median Force | — | — | — | -27.53 | 53.83 | 0.61 |
| $R^{2}$ | 0.06 | | | 0.30 | | |
| LR test^d^: $\chi^{2}$ | 32.91 | | | | | |
| LR test: P value | .001 | | | | | |

^a^ Definitions of gait features in this table are shown in Table 1.

^b^ SE: standard error.

^c^ Not applicable.

^d^ The critical value of the likelihood ratio statistic: $\chi_{0.05}^{2}$(12) = 21.03.
